# Study on Properties Prediction and Braiding Optimization of Axial Braided Carbon/Carbon Composite

**DOI:** 10.3390/ma13112588

**Published:** 2020-06-05

**Authors:** Chunguang Wang, Peng Cao, Min Tang, Weiping Tian, Ketong Liu, Baorui Liu

**Affiliations:** 1 College of Electrical and Control Engineering, Shaanxi University of Science & Technology, Xi’an 710021, China; 2 College of Architecture and Civil Engineering, Beijing University of Technology, Beijing 100124, China; caopeng518888@126.com; 3 The Fourth Academy of CASC, Xi’an 710025, China; leegoop@126.com (M.T.); chencong0269@163.com (W.T.); 4 College of Architecture and Civil Engineering, Xi’an University of Science and Technology, Xi’an 710054, China; ketong-1982@163.com; 5 Science and Technology on Reliability and Environment Engineering Laboratory, Beijing Institute of Structure and Environment Engineering, Beijing 100076, China; wolfman85@163.com

**Keywords:** axial braided C/C composites, macroscopic properties, prediction method, microstructure characteristics, braiding spacing

## Abstract

It is well established that the microstructure has significant effects on the properties of axial braided C/C composites. In this study, a method coupling the homogenization method and finite element method (FEM) was proposed to predict the relationship between the microstructure characteristics and macroscopic properties. Based on the representative volume element (RVE) model, the periodic displacement boundary condition was introduced to predict the equivalent elastic properties of the RVE and component of C/C composite material, and the coefficient of thermal expansion (CTE) of the material was predicted by the energy prediction method. The predicted results were in good agreement with experimental results. By predicting the thermal and mechanical properties of the materials with different braiding spacing and fiber rod diameter, the variation of the properties with braiding spacing and fiber rod diameter was obtained. The research methods and results in this paper could provide important references for the optimization and rational application of composite materials.

## 1. Introduction

Axial braided C/C composite is a special carbon fiber reinforced carbon matrix composite. Both the reinforcing phase and matrix phase are composed of pure carbon [1,2,3,4,5]. It exhibits many superior properties, such as high specific strength, good thermal stability, wear resistance and ablative resistance. Specially, different from common materials, its mechanical properties do not decrease but rather increase with the increase of temperature, which makes it widely used in aviation and aerospace fields [6,7]. Due to its complex internal microstructures, such as porosity, cracks, pore mean and pore shape, it is difficult to quantify the effects of microstructure characteristics on its macroscopic properties based on experiments. Therefore, numerical and theoretical methods calibrated and verified by experiments are required to evaluate the relationship between its microstructure characteristics and macro properties [8,9,10,11,12].

Many methods have been introduced to predict the mechanical properties of braided C/C composites [13,14,15,16]. Among them, the stiffness performance has been well investigated. With the development of finite element method (FEM), the method for predicting the properties based on the homogenization theory provides a new idea for predicting the properties of C/C composite materials. Because the FEM is used in this method, the microstructure characteristics and distribution in the fiber rod (bundle) can be considered. Because the homogenization theory is combined, the interaction between components can be taken into account in the model, so that the stiffness prediction accuracy of C/C composite materials is higher [17,18,19]. The energy method-based prediction method of CTE of composite materials derived by Zhang [20,21,22,23] is more convenient to calculate than the homogenization method, and has been gradually popularized in the industry.

In this paper, the braided structure characteristics of axial braided C/C composites were considered, the homogenization method was combined with FEM by ABAQUS. Besides, the energy method was introduced to establish the prediction method of macroscopic properties of materials. A Python program was used to generate the matrix, interface and fiber rod (bundle) geometric models that meet the distribution law of microstructure characteristics and predict their stiffness. The predicted results and the measured results were taken as the input of FEM. Based on the RVE model of materials, periodic displacement boundary conditions were introduced to predict the equivalent elastic properties and the CTE of axial braided C/C composites [17,18,19,20]. By predicting the macroscopic properties of the material with different braiding spacing and fiber rod diameter, the variation of material properties was obtained, which provided a reference for the optimization and rational application of the material.

## 2. A Method for Predicting the Macroscopic Properties of Axial Braided C/C Composite

### 2.1. Homogenization Process and Stiffness Prediction Method

**Figure 1:**
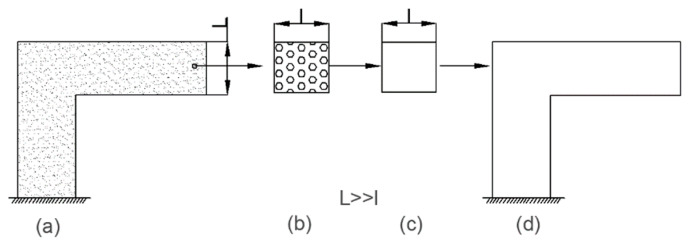
Composite structure, representative volume element and equivalent structure. (**a**) original structure (**b**) micro element (**c**) homogenized element (**d**) homogenized structure.

The homogenization theory has been widely recognized to predict the stiffness of composites, and its concept is shown in Figure 1. In order to solve the difficulty of large-scale macro-micro joint computation, the concept of microelement is introduced. A microelement represents a point in the composite structure, and its average stress and strain relation is regarded as the effective constitutive relation of composite. In fact, the non-uniform element is replaced by a representative volume element (RVE) with the above average stress and strain relationship. The above concepts are applied to every point of the composite structure. Based on such way, the original problem of analyzing the non-uniform composite structure was converted to the problem of analyzing the properties of the macro structure after homogenization, which greatly simplified the original problem [24,25].

**Figure 2:**
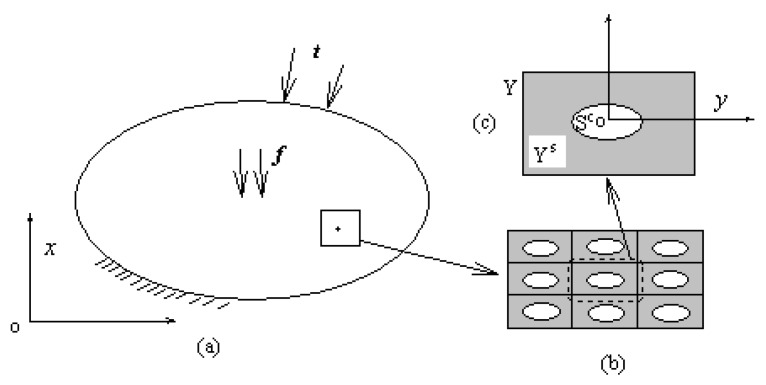
Composite material with mesoscopic structure. (**a**) Macro structure (**b**) Mesoscopic structure and its periodicity (**c**) RVE.

Meso-level composites often exhibit a high degree of heterogeneity, and even in the neighborhood ε with a very small macroscopic position x, the field variables will vary greatly, as shown in Figure 2. 

Considering the periodicity of the composite structure, these field variables can be expressed as follows:

(1) $$\Phi^{\varepsilon }\left(x\right)=\Phi \left(x,y+kY\right),y=x/\varepsilon ,\frac{\partial \phi^{\varepsilon }}{\partial x}\;=\;\frac{\partial \phi }{\partial x}+\frac{1}{\varepsilon }\frac{\partial \phi }{\partial y}$$

where, x is the macroscopic scale, y is the mesoscopic scale, ε is the ratio of the two scales, Y is the period of the periodic function, and k is the integer.

The displacement is expressed as a function of double-scale coordinates and expanded according to small parameters:

(2) $$u^{\varepsilon }\left(x\right)=\left(x,y\right)+\varepsilon^{1}u^{1}\left(x,y\right)+\varepsilon^{2}u^{2}\left(x,y\right)+\cdot \cdot \cdot ,\;y=x/\varepsilon$$

$u^{\left(i\right)}\left(x,y\right)$ is a periodic function of Y. According to constitutive equation and geometric equation in elastic mechanics, the above asymptotic expansion was substituted into the equilibrium equation, and the left side of the equation was arranged as an asymptotic expansion with respect to ε. We set the coefficient of $\varepsilon^{i}\left(i=-1,0,1,\cdots \right)$ as zero to obtain a series of governing equations, in which:

(3) $$\frac{\partial \sigma_{ij}^{\left(-1\right)}\left(x,y\right)}{\partial y_{j}}\;=0$$

(4) $$\frac{\partial \sigma_{ij}^{\left(0\right)}\left(x,y\right)}{\partial y_{j}}+\frac{\partial \sigma_{ij}^{\left(-1\right)}\left(x,y\right)}{\partial x_{j}}=0$$

In Equation (4), $\sigma_{ij}\left(x,y\right)$ is a function of stress field. By simplifying the operation, the following equation was obtained:

(5) $$\sigma_{ij}^{\left(0\right)}=\left(C_{ijkl}-C_{ijmn}\partial \chi_{m}^{kl}/\partial y_{n}\right)\varepsilon_{xkl}\left(u^{\left(0\right)}\right)$$

(6) $${\left[C_{ijkl}\left(\chi_{\left(k,y_{l}\right)mn}+I_{klmn}\right)\right]}_{y_{j}}=0\;\;\mathrm{on}\;Y$$

(7) $$\chi_{imn}\left(y\right)=\chi_{imn}\left(y\;+Y\right)\;\;\mathrm{on}\;\partial Y$$

(8) $$\chi_{imn}\left(y\right)=0\;\;\mathrm{on}\;\partial Y^{\mathrm{vert}}$$

Among them:

(9) $$I_{klmn}=\left(\delta_{mk}\delta_{nl}+\delta_{nk}\delta_{ml}\right)/2$$

(10) $$\chi_{\left(k,y_{l}\right)mn}=\frac{1}{2}\left(\frac{\partial \chi_{kmn}}{\partial y_{l}}+\frac{\partial \chi_{lmn}}{\partial y_{k}}\right)$$

The periodic function Y can be expressed as the region of a single cell in the axial braided carbon/carbon composite. ∂Y is the boundary of the cell, and $\partial Y^{\mathrm{vert}}$ is the vertex of the cell on the boundary. The homogenization coefficient of a single cell can be expressed as:

(11) $$C_{ijkl}^{H}=\frac{1}{\left|Y\right|}\int_{Y}C_{ijkl}(\chi_{\left(k,y_{l}\right)mn}+I_{klmn})dV$$

$\sigma^{\left(0\right)}\left(x,y\right)$ is the second term of the asymptotic expansion of $\sigma^{\varepsilon }\left(x,y\right)$, which is called the mesoscopic stress field. The mesoscopic stress field includes the macroscopic parameters x and the mesoscopic parameters y, which reflect the fluctuation of stress in the scale of RVE.

Gauss theorem is used to treat the governing equation of the homogenization coefficient of Equation (11), and the equation is converted into a “weak” solution equivalent to the original problem as follows:

(12) $$\int_{Y}C_{ijkl}\chi_{\left(k,y_{l}\right)mn}\;\frac{1}{2}\left(\frac{\partial \delta v_{i}}{\partial y_{j}}+\frac{\partial \delta v_{j}}{\partial y_{i}}\right)dV=-\underset{p}{\sum }\int_{\partial Y^{p}-\partial Y^{p}\cap \partial Y}C_{ijmn}n_{j}\delta v_{i}dS$$

From Equation (12) can be found in the original control equation is equivalent to the three dimensional linear elastic problems in the form of the "weak" solution of equation, the difference is that the material within a plane distribution related to the material properties and boundary shape, the plane distribution of force on the internal boundary of different material in the RVE ($\partial Y^{p}-\partial Y^{p}\cap \partial Y$), pointing to the internal material area, the direction of the i component for $C_{ijmn}n_{j}$.

The two terms in the integral sign of Equation (12) were separated to obtain the deformation of the homogenization coefficient:

(13) $$C_{ijkl}^{H}=\frac{1}{\left|Y\right|}\int_{Y}C_{ijkl}\chi_{\left(k,y_{l}\right)mn}dV+\frac{1}{\left|Y\right|}\int_{Y}C_{ijmn}dV$$

From Equation (13), it can be seen that in the boundary force method, the homogenization coefficient is equal to the volume average of the stress of the three-dimensional linear elastic problem on the RVE plus the volume average of the elastic tensor on the RVE [16,17,18]. The homogenization coefficient is the effective elastic property of macroscopic structure.

### 2.2. A Method for Predicting CTE of Composites Based on Energy Method

The CTE prediction method based on the energy method is more convenient to calculate than the simple homogenization method. Therefore, the following method combined with FEM was used to calculate the CTE of materials in this paper [19,22].

The basic idea of the energy method is that based on the relationship between microstructure and homogeneous equivalent body, the energy expression of the equivalent property of composite materials can be obtained by deducing the relationship between the equivalent property of composite materials and the deformation energy of microstructure [16,17].

For the microstructure shown in Figure 1, when the temperature rise value is ΔT, the average stress and average strain of the microstructure have the following relationship:

(14) $$\bar{\sigma }=C^{H}\left(\bar{\varepsilon }-\alpha^{H}\Delta T\right)$$

where $C^{H}$, $\alpha^{H}=\left[\alpha_{11}^{H},\alpha_{22}^{H},\alpha_{33}^{H}\right]$ are the stiffness matrix and CTE matrix of axial braided C/C composites.

The deformation energy of the microstructure of the material is shown in Equation (15):

(15) $$C=\int_{\Omega }\varepsilon^{T}D\varepsilon d\Omega$$

**Figure 3:**
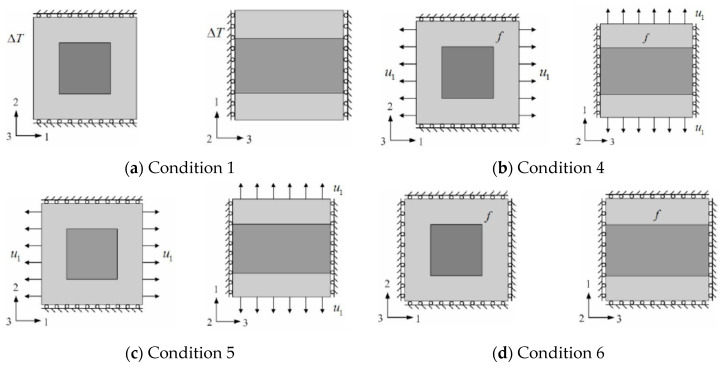
Microstructure forms under different operating conditions. Among of them, working condition 2 and working condition 3 are similar to working condition 1, which are not completely shown in the figure.

For working condition 1 ($u_{2}=u_{3}=0$) as shown in Figure 3a, the microstructure deformation energy caused by ΔT temperature change is the same as that of working condition 4 without temperature load as shown in Figure 3b. The displacement load $u_{1}$ in working condition 4 is equal to the displacement load under working condition 1. The force f is the interface force caused by the difference of thermal stress between the two loads. The expressions of the two are Equations (16) and (17).

(16) $$u_{1}=\left(\alpha_{11}^{H}\Delta T+\left(D_{1122}^{H}/D_{1111}^{H}\right)\alpha_{22}^{H}\Delta T+\left(D_{1133}^{H}/D_{1111}^{H}\right)\alpha_{33}^{H}\right)l$$

(17) $$f=\int_{s}\left[D\left(E_{1}\right)\alpha \left(\alpha_{1}\right)-D\left(E_{2}\right)\alpha \left(\alpha_{2}\right)\right]\Delta Td_{s}$$

For the microstructure of working condition 1, the variable properties of its homogeneous equivalent are equal to the difference between the variable properties of working condition 1 and that of working condition 6, as shown in Equation (18):

(18) $$C_{em}^{\left(1\right)}=C_{m}^{\left(1\right)}-C_{m}^{\left(6\right)}=D_{1111}^{H}{\left(\alpha_{11}^{H}\Delta T+\left(D_{1122}^{H}/D_{1111}^{H}\right)\alpha_{22}^{H}\Delta T+\left(D_{1133}^{H}/D_{1111}^{H}\right)\alpha_{33}^{H}\Delta TV\right)}^{2}$$

Similar to Equation (18), the variable properties of the homogeneous equivalent of condition 2 ($u_{1}=u_{3}=0$) and condition 3 ($u_{1}=u_{2}=0$) can be expressed as Equations (19) and (20):

(19) $$\begin{array}{c} C_{em}^{\left(2\right)}=C_{m}^{\left(2\right)}-C_{m}^{\left(6\right)}=D_{2222}^{H}{\left(\alpha_{22}^{H}\Delta T+\left(D_{1122}^{H}/D_{2222}^{H}\right)\alpha_{11}^{H}\Delta T+\left(D_{2233}^{H}/D_{2222}^{H}\right)\alpha_{33}^{H}\Delta T\right)}^{2}\\ C_{em}^{\left(3\right)}=C_{m}^{\left(3\right)}-C_{m}^{\left(6\right)}=D_{3333}^{H}{\left(\alpha_{33}^{H}\Delta T+\left(D_{1133}^{H}/D_{3333}^{H}\right)\alpha_{11}^{H}\Delta T+\left(D_{2233}^{H}/D_{3333}^{H}\right)\alpha_{22}^{H}\Delta T\right)}^{2} \end{array}$$

The FEM equation of the microstructure under operating conditions 4, 5 and 6 can be expressed as Equation (20).

(20) $$\left[\begin{array}{cc} k_{11} & k_{12}\\ k_{21} & k_{22} \end{array}\right]\left[\begin{array}{c} U_{1}^{\left(i\right)}\\ U_{2}^{\left(i\right)} \end{array}]=[\begin{array}{c} F_{1}^{\left(i\right)}\\ F_{2}^{\left(i\right)} \end{array}\right]\;i=4,5,6$$

where, $U_{1}^{\left(i\right)}$ is the unknown node displacement vector under condition i, and $U_{2}^{\left(i\right)}$ is the known node displacement vector. $F_{1}^{\left(i\right)}$ is the known node force vector under condition i, and $F_{2}^{\left(i\right)}$ is the unknown node force vector. To solve Equation (20), the following Equation (21) can be obtained.

(21) $$\begin{array}{c} U_{1}^{\left(i\right)}=K_{11}^{-1}F_{1}^{\left(i\right)}-K_{11}^{-1}K_{12}U_{2}^{\left(i\right)}\\ F_{2}^{\left(i\right)}=K_{21}K_{11}^{-1}F_{1}^{\left(i\right)}+\left(K_{22}-K_{21}K_{11}^{-1}K_{12}\right)U_{2}^{i} \end{array}$$

The microstructural variable performance of working condition i can be expressed in the following form:

(22) $$C_{m}^{\left(i\right)}=\int_{\Omega }\varepsilon^{(i)T}D\varepsilon^{\left(i\right)}d_{\Omega }=F_{1}^{(i)T}U_{1}^{\left(i\right)}+F_{2}^{(i)T}U_{2}^{\left(i\right)}$$

The calculation method of CTE of microstructure can be obtained by solving Equation (22) as shown in Equation (23):

(23) $$\begin{array}{l} \alpha_{11}^{H}=\frac{\left(\frac{D_{1122}^{H}}{D_{1111}^{H}}\frac{D_{2233}^{H}}{D_{2222}^{H}}-\frac{D_{1133}^{H}}{D_{1111}^{H}})(\frac{D_{2233}^{H}}{D_{3333}^{H}}Q-R)-(\frac{D_{2233}^{H}}{D_{3333}^{H}}\frac{D_{2233}^{H}}{D_{2222}^{H}}-1)(\frac{D_{1122}^{H}}{D_{1111}^{H}}Q-P\right)}{\left(\frac{D_{1122}^{H}}{D_{1111}^{H}}\frac{D_{2233}^{H}}{D_{2222}^{H}}-\frac{D_{1133}^{H}}{D_{1111}^{H}})(\frac{D_{2233}^{H}}{D_{3333}^{H}}\frac{D_{1122}^{H}}{D_{2222}^{H}}-\frac{D_{1133}^{H}}{D_{3333}^{H}})-(\frac{D_{2233}^{H}}{D_{3333}^{H}}\frac{D_{2233}^{H}}{D_{2222}^{H}}-1)(\frac{D_{1122}^{H}}{D_{1111}^{H}}\frac{D_{1122}^{H}}{D_{2222}^{H}}-1\right)}\\ \alpha_{22}^{H}=\frac{\left(\frac{D_{1122}^{H}}{D_{2222}^{H}}\frac{D_{1133}^{H}}{D_{1111}^{H}}-\frac{D_{2233}^{H}}{D_{2222}^{H}})(\frac{D_{1133}^{H}}{D_{3333}^{H}}P-R)-(\frac{D_{1133}^{H}}{D_{3333}^{H}}\frac{D_{1133}^{H}}{D_{1111}^{H}}-1)(\frac{D_{1122}^{H}}{D_{2222}^{H}}P-Q\right)}{\left(\frac{D_{1122}^{H}}{D_{2222}^{H}}\frac{D_{1133}^{H}}{D_{1111}^{H}}-\frac{D_{2233}^{H}}{D_{2222}^{H}})(\frac{D_{1133}^{H}}{D_{3333}^{H}}\frac{D_{1122}^{H}}{D_{1111}^{H}}-\frac{D_{2233}^{H}}{D_{3333}^{H}})-(\frac{D_{1133}^{H}}{D_{3333}^{H}}\frac{D_{1133}^{H}}{D_{1111}^{H}}-1)(\frac{D_{1122}^{H}}{D_{2222}^{H}}\frac{D_{1122}^{H}}{D_{1111}^{H}}-1\right)}\\ \alpha_{33}^{H}=\frac{\left(\frac{D_{1133}^{H}}{D_{3333}^{H}}\frac{D_{1122}^{H}}{D_{1111}^{H}}-\frac{D_{2233}^{H}}{D_{3333}^{H}})(\frac{D_{2233}^{H}}{D_{3333}^{H}}P-Q)-(\frac{D_{1122}^{H}}{D_{2222}^{H}}\frac{D_{1122}^{H}}{D_{1111}^{H}})-1)(\frac{D_{1133}^{H}}{D_{3333}^{H}}P-R\right)}{\left(\frac{D_{1133}^{H}}{D_{3333}^{H}}\frac{D_{1122}^{H}}{D_{1111}^{H}}-\frac{D_{2233}^{H}}{D_{3333}^{H}})(\frac{D_{1122}^{H}}{D_{2222}^{H}}\frac{D_{1133}^{H}}{D_{1111}^{H}}-\frac{D_{2233}^{H}}{D_{2222}^{H}})-(\frac{D_{1122}^{H}}{D_{2222}^{H}}\frac{D_{1122}^{H}}{D_{1111}^{H}})-1)(\frac{D_{1133}^{H}}{D_{3333}^{H}}\frac{D_{1133}^{H}}{D_{1111}^{H}}-1\right)} \end{array}$$

In the equation $P=\sqrt{\frac{C_{m}^{\left(1\right)}-C_{m}^{\left(6\right)}}{D_{1111}^{H}{\left(\Delta T\right)}^{2}V}},Q=\sqrt{\frac{C_{m}^{\left(2\right)}-C_{m}^{\left(6\right)}}{D_{2222}^{H}{\left(\Delta T\right)}^{2}V}},R=\sqrt{\frac{C_{m}^{\left(3\right)}-C_{m}^{\left(6\right)}}{D_{3333}^{H}{\left(\Delta T\right)}^{2}V}}$.

### 2.3. The Boundary Conditions

According to the braided characteristics of the axial braided C/C composites studied in this paper, each surface of the RVE model must be set as a periodic symmetric boundary condition. The displacement of the RVE should also meet the periodic boundary conditions [17]. For the periodic RVE structure with spatial translational symmetry, the periodic condition of its displacement can be expressed as follows:

(24) $$\begin{array}{c} u_{2}\left(y_{1}^{0},y_{2},y_{3}\right)=u_{1}\left(y_{1}^{0}+Y_{1},y_{2},y_{3}\right)\\ u_{2}\left(y_{1},y_{2}^{0},y_{3}\right)=u_{1}\left(y_{1},y_{2}^{0}+Y_{2},y_{3}\right)\\ u_{2}\left(y_{1},y_{2},y_{3}^{0}\right)=u_{1}\left(y_{1},y_{2},y_{3}^{0}+Y_{3}\right) \end{array}$$

## 3. The Introduction of the Experiment Carried out in the Early Stage

### 3.1. Properties Test of Fiber Rod

Under the premise of minimum damage, the z-direction fiber rod (XY direction is transverse) was stripped, and the tensile and compression properties of the fiber rod were tested to obtain its stiffness properties. A special fixture was designed for its test. In the test, the unconstrained connection mechanism was adopted to eliminate the error caused by eccentricity when the sample was loaded. The accurate strain of fiber rods was obtained by non-contact optical strain testing system (ARAMIS, 2.3M, GOM, Brunswick, Germany). The obtained stiffness properties of the fiber rod are shown in Table 1. 

### 3.2. Macroscopic Properties Test

The macroscopic mechanical properties of axial braided C/C composites mainly include uniaxial tensile test and uniaxial compression test. Samples under different loading conditions were designed according to the braiding mode and product size of the axial braided C/C composite. Samples with different directions including fiber rod direction (axial direction) and fiber bundle direction (radial direction) were tested and more than 10 samples were prepared for each group. The exact strain of specimens was obtained by non-contact optical strain testing system (ARAMIS). The stiffness properties of axial braided C/C composites are shown in Table 2.

## 4. RVE Establishment and Properties of Component 

### 4.1. RVE Establishment

**Figure 4:**
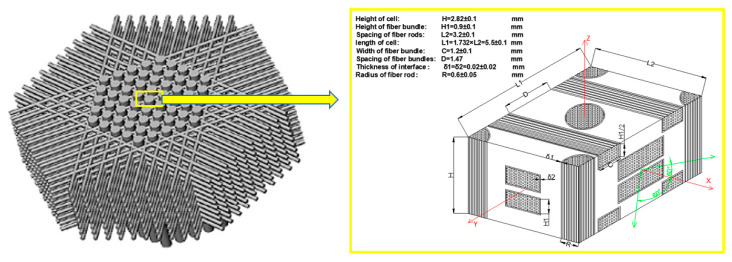
RVE of axial braided C/C composite.

The RVE of axial braided C/C composite material is a rectangular structure composed of fiber rod, fiber bundle, matrix and interface. The braided structure and phase scale of each component are shown in Figure 4. Thin carbon fiber rigid rod formed by pultrusion forms the axial reinforcing network, and the soft carbon fiber bundle is woven into the pre-texture. The fiber rods are arranged in an equilateral triangle in the axial direction, and the fiber bundles are successively increased through the 0°, 60° and 120° channels of cambium formed by the fiber rods, and so on until the required size of pre-texture is formed. High density 4D C/C composites were prepared by asphalt impregnation, carbonization, densification and high temperature treatment. The minimum unit of this pre-texture is axially symmetric, and its braided thickness accumulates axially, so it is called axial braided C/C composite.

**Figure 5:**
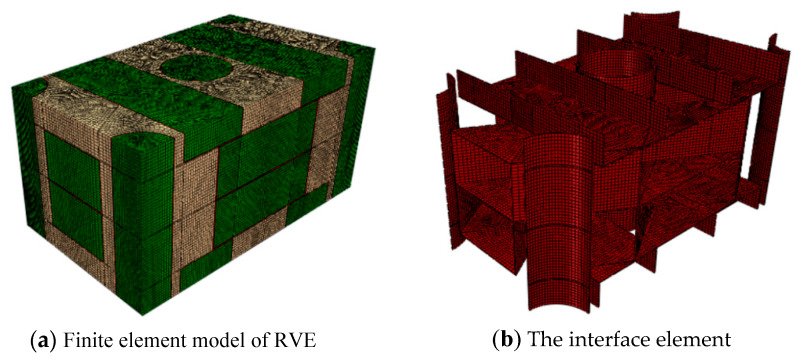
Finite element model of axial braided C/C composite material.

The generated finite element model of axial braided C/C composite material composed from the RVE model is shown in Figure 5. The division of the grid is highly related to the application of periodic boundary conditions, so it is necessary to strictly guarantee the uniformity of the grid nodes on the corresponding surface of RVE. In this paper, C3D8 elements and C3D6 elements were used to mesh the fiber rod, fiber bundle and matrix. The fiber rod/matrix interface and fiber bundle/matrix interface were all meshed by C3D8 elements [24,25,26,27,28].

### 4.2. Study on the Properties of Components

The premise of RVE model performance calculation with finite element technique is that the properties of each meso components (including the fiber rod and bundle, matrix and interface) are known. Due to the poor stripping and testability of the meso components, some of the necessary properties need to be obtained in combination with numerical calculations.

#### 4.2.1. Mechanical Properties of Matrix and Interface

The pores and cracks in the matrix and interface of the axial braided C/C composite were regarded as inclusions with different shapes and sizes and introduced into the pure carbon matrix. In this paper, the random geometric models meeting objective distribution law of microstructure characteristics were generated by a subroutine coded by python and its mechanical properties were calculated by FEM, which can consider the interaction between various microstructures [28,29,30,31,32].

**Figure 6:**
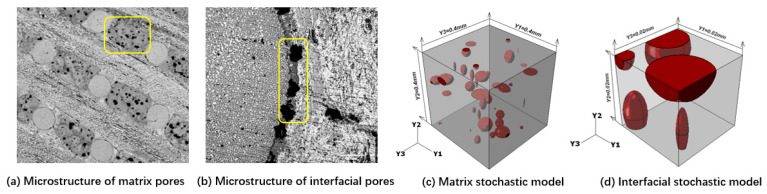
Computational model of matrix and interface.

In order not only to simplify the calculation workload but also to contain sufficient pore information, the side length of the calculation model of the matrix was selected as 0.4 mm, and the side length of the interface was selected as 0.02 mm, and the generated model was shown in Figure 6. In the model, the pores are mainly ellipsoid, and the ratio of long axis and short axis of ellipsoid at some locations is large, resulting in cracks. In the early stage, the non-pore matrix modulus was 12.34 GPa and Poisson’s ratio was 0.23 by using nano-indentation experiment [16,17], which was used as the material input for finite element calculation.

**Figure 7:**
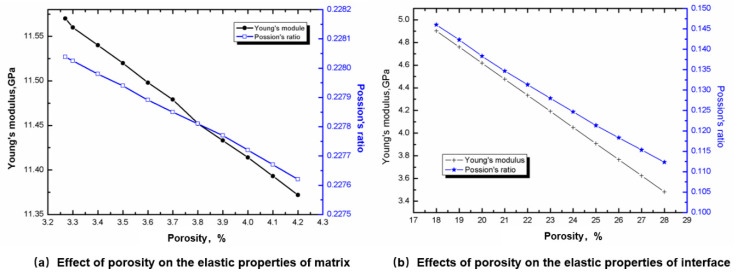
Effect of porosity on the elastic properties of matrix and interface.

Figure 7a shows the effect of porosity on the elastic properties of the matrix. It can be seen that the elastic properties of the matrix vary linearly with porosity. The increase of porosity leads to the decrease of both elastic modulus and Poisson’s ratio.

Figure 7b shows the influence of porosity on the interface elastic properties. It can be found that the influence trend of porosity on the interface elastic properties is the same as that of porosity on the matrix.

Based on the statistical study in [2], the porosity of the matrix was 0.036, and the modulus and Poisson’s ratio were 11.5 GPa/0.228 respectively, obtained from Figure 7a. Based on the statistical study in [2], the porosity of the interface was 0.27, and the modulus and Poisson’s ratio were 3.91 GPa/0.12, respectively, obtained from Figure 7b. The CTE of the matrix was obtained from the preliminary test results, −1.3 × 10^−6^/K.

#### 4.2.2. Study on Mechanical Properties of Fiber Rod and Fiber Bundle

**Figure 8:**
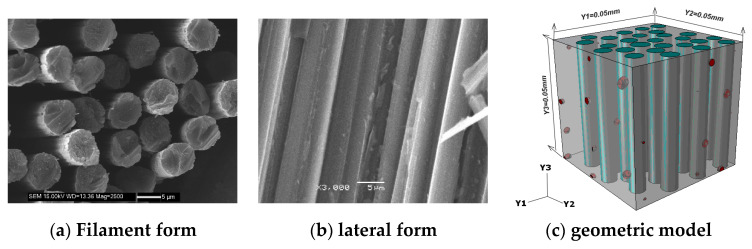
SEM morphology and geometric model of fiber rod (bundle).

In this section, based on the microstructure characteristics and random distribution of fiber rod (bundle), FEM and Python language programming were used to generate the fiber rod (bundle) geometric model satisfying the distribution law of microstructure characteristics, and the homogenization method was used to predict the stiffness properties. Figure 8 shows the microscopic observation image and geometric model of the fiber rod. The model consists of fiber monofilament, matrix and pores.

Based on relevant experiments and combined with literature [16,17], the input parameters of fiber monofilament and matrix were determined as shown in Table 3. According to the literatures, these data were directly measured by the experiment. 

The stiffness properties of the reinforcing phase were calculated by FEM. The results show that the reinforcing phase was transversally isotropic even if there are a few pores. Table 4 lists the calculation results of the enhancement phase and some experimental results in the early stage. It can be seen from the table that the prediction error of fiber rod’s tensile modulus, compression modulus and shear modulus is 13.4%, 13.7% and 10.4% respectively, which verifies the reliability of the calculation method for the reinforcement phase. There are two reasons for the error: the error of the test system and the test method; The inaccuracy of porosity observation leads to the inaccuracy of FEM. In the subsequent calculations of the macroscopic properties of the material, the longitudinal properties of the rod were given according to the experimental values, while the shear properties and other untested parameters were given according to the predictive values.

## 5. Calculation Results and Analysis

### 5.1. The Calculation Results of Elastic Properties

Periodic boundary conditions were applied to the RVE model of the axial C/C composite material to obtain its effective properties as shown in Table 5. By comparing the prediction results with the experimental results under different conditions, it can be found that the elastic modulus of the numerical prediction results is basically consistent with the experimental results, thus verifying the effectiveness of the prediction method.

### 5.2. The Calculation Result of CTE

Based on the material parameters listed in Table 3 and Table 4, the energy analysis method was used to predict the CTE of the RVE model. According to previous research experience, the effect of porosity on CTE is very weak, so this paper did not study the effect of porosity on CTE. The comparison between the predicted results and the experimental results is shown in Table 6. The experimental data of thermal expansion in the table were tested by DIL 402 PC (NETZSCH, Lanzhou, China), and the sample size was φ6 mm × 50 mm.

The comparison between the results of prediction and experimental results shows that the prediction method based on energy method has certain prediction accuracy. However, the predicted value in both directions is larger than the experimental value, which may be caused by the fact that the CTE of the matrix is larger than the real value.

### 5.3. Effect of Braiding Spacing on Stiffness and CTE

**Figure 9:**
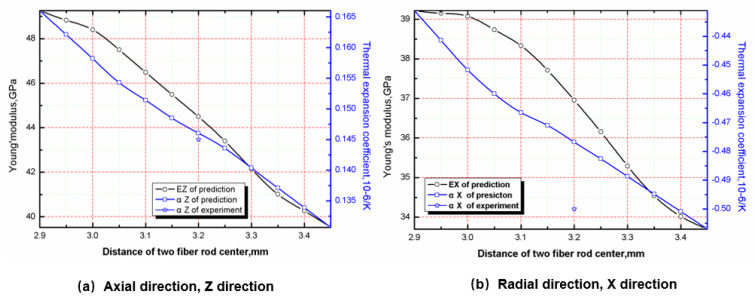
Effect of center spacing of fiber rod on axial and radial tensile modulus and CTE.

The stiffness and CTE of the material can be predicted by changing the center spacing of the fiber rod. The influence of braiding spacing on the tensile modulus and CTE is shown in Figure 9. With the increase of braiding spacing, the tensile modulus and CTE in axial and radial directions decrease gradually. The main reason is that the volume content of axial fiber rod and radial fiber bundle in RVE decreases while the volume fraction of matrix increases with the increase of braiding distance. Since the modulus and CTE of the matrix are lower than that of the fiber rod and fiber bundle, the increase of the volume fraction will inevitably lead to the overall decrease of the modulus and CTE [28,29,30,31,32,33].

From the perspective of the use environment of C/C composite materials, it is necessary to reduce the modulus and CTE to reduce the thermal stress of the structural parts. Therefore, the increase of braiding spacing of materials is more in line with the requirements. However, studies have shown that the fiber volume fraction is closely related to the ablative resistance of the material. When the fiber volume fraction decreases, the ablative rate of the material increases, which is unfavorable for engineering application. Therefore, the optimization of braiding spacing of C/C composites should be combined with the ablative properties of the materials. The influence of braiding spacing on ablation rate will be studied in the future.

### 5.4. Effect of Fiber Rod Diameter on Stiffness and CTE

**Figure 10:**
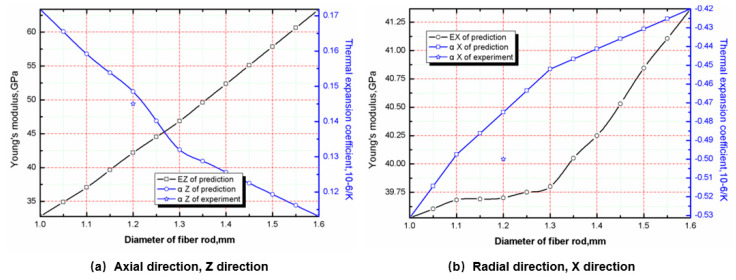
Effect of fiber rod diameter on radial tensile modulus and CTE of axial braided C/C composites.

The influence of fiber rod diameter on tensile modulus and the CTE is shown in Figure 10a. As can be seen, with the increase of fiber rod diameter, the axial tensile modulus increases significantly, while the CTE decreases significantly. This is mainly because, under the condition of constant braiding spacing, the diameter of fiber rod increases, which significantly increases the content of axial fiber in RVE. In addition, due to the higher modulus of axial fiber and the lower CTE, the axial modulus of the material increases and the CTE decreases.

Radial tensile modulus and CTE are both incremental functions of fiber rod diameter, but the increment is small, as shown in Figure 10b.The reason is that for the radial tension modulus, when the braiding spacing is constant, increasing the diameter of the fiber rod will not cause the change of the volume fraction of the radial fiber bundle without changing the fiber bundle’s own size, but will cause the decrease of the volume fraction of the matrix. Because the modulus of the matrix is smaller than the transverse modulus of the fiber rod, the radial tensile modulus increases. For the CTE, since the CTE of the matrix is smaller than transverse CTE of the fiber rod, when the volume fraction of the fiber rod increases and the volume fraction of the matrix decreases, the increase of transverse CTE of the fiber rod to the radial CTE of the material is greater than the decrease of radial CTE of the matrix. Figure 10b shows that it is appropriate for the diameter of the axial braided C/C composite fiber rod to be around 1.26 mm.

## 6. Conclusions

In this paper, the stiffness prediction method based on homogenization method and the CTE prediction method based on energy method were established. In the prediction of component stiffness properties, Python language was used to generate a component material model that meets the characteristics of pore microstructure randomly, so that the prediction of component material properties was more accurate. The following conclusions are obtained:

(1) The FEM was utilized for the calculation of the stiffness properties of the matrix and interface, and the results showed that the porosity was important to determine the elastic properties of the matrix and interface. It can be seen that the elastic properties of the matrix and interface change linearly with the porosity, which increases, leading to decreases of both the elastic modulus and Poisson’s ratio.
(2) The stiffness performance of the reinforcing phase was calculated by FEM. The calculation results showed that the reinforcing phase manifested transversely isotropic characteristic even if there was a few of pores. It was found that the prediction precision of tensile modulus, compression modulus and shear modulus was reliable, being 13.4%, 13.7% and 10.4%, respectively.
(3) Based on the homogenization theory and energy method combined with the RVE of axial braided C/C composite material, the macroscopic stiffness and CTE of the material were also predicted. By comparing the predicted results with the experimental results in different states, it can be concluded that the numerical predicted results are basically consistent with the experimental results, thus proving the effectiveness of the prediction method.
(4) By calculating the macroscopic stiffness performance and CTE under the conditions with different braiding spacing and fiber rod diameter, the relationship between the microstructure and macroscopic properties was evaluated. The results show that the best diameter of the axial braided C/C composite fiber rod studied in this paper is 1.26 mm, and the optimization of braiding spacing needs to be carried out in combination with the ablative property.
(5) The advantages of the prediction method in this paper are as follows: by combining the homogenization method with FEM, the pore characteristics of the fiber rod (bundle), matrix and interface can be considered, so as to obtain the properties of mesoscopic materials accurately. As the input parameters, the stiffness prediction of the material has higher precision. The disadvantage is that the pore characteristics cannot be added into the finite element model of RVE for the joint calculation of macroscopy-mesoscopic and microscopic, which is also the focus of the follow-up research of this project.

## Figures and Tables

**Table 1:** Stiffness characteristics of fiber rod.

Item	*E_x_*, GPa		$\nu_{xy}=\nu_{xz}$	
	Mean Value	Standard Deviation	Mean Value	Standard Deviation
Tension	211.1	10.3	0.06	0.02
Compression	99.4	8.47	0.15	0.03

Note: Angle mark *x* represents the fiber rod axial direction, Angle mark *y* and *z* represent the transverse direction.

**Table 2:** Stiffness characteristics of axial braided C/C composites.

Item	$E_{X},GPa$ ($G_{XY},\mathrm{GPa}$)		$\nu_{XY}$		$E_{Z},\mathrm{GPa}$ ($G_{XZ},\mathrm{GPa}$)		$\nu_{XZ}$	
	Mean Value	Standard Deviation	Mean Value	Standard Deviation	Mean Value	Standard Deviation	Mean Value	Standard Deviation
Tension	38.3	2.3	0.28	0.41	40.5	4.2	0.09	0.05
Compression	20.9	1.9	0.28	0.04	22.1	2.1	0.09	0.05
Shear	12.6	1.3			3.4	0.5		

Note: Angle mark *Z* represents the fiber rod axial direction, Angle mark *X* and *Y* represent the transverse direction.

**Table 3:** Properties of fiber and matrix in reinforcing phase.

Item	$E_{x},\mathrm{GPa}$	$E_{y},\mathrm{GPa}$	$G_{xy},\mathrm{GPa}$	$\nu_{xy}$	$\nu_{yz}$	α_x_, 10^−6^/K	α_y_ = α_z_, 10^−6^/K
T300 fiber	345.1☆120.0★	19.0	26.7	0.2	0.35	−0.78	8.14
Matrix	12.34	-	-	0.23	-	−1.3	−1.3

Note: ☆ represents the tensile modulus. ★ represents the compression modulus. Angle mark *x* represents the fiber filaments axial direction, Angle mark *y* and *z* represent the transverse direction. The parameters of the matrix are the properties without pores.

**Table 4:** Stiffness properties of the enhanced phase.

Item	$E_{x},\mathrm{GPa}$	$E_{y},\mathrm{GPa}$	$G_{xy},\mathrm{GPa}$	$\nu_{xy}$	$\nu_{yz}$	α_x_, 10^−6^/K	α_y_ = α_z_, 10^−6^/K
The predictive value of the fiber rod	239.5☆85.8★	17.37	20.25	0.11	0.45	−0.94	5.1
Test value of fiber rod	211.1☆99.4★		22.6	0.06	-	-	-
The predictive value of fiber bundle	220.8☆79.1★	17.11	18.96	0.12	0.40	−0.99	4.52

Note: ☆ represents the tensile modulus. ★ represents the compression modulus. Angle mark *x* represents the fiber rod (bundle) axial direction, Angle mark *y* and *z* represent the transverse direction.

**Table 5:** Mechanical properties of axial braided C/C composites.

Item	Elastic Modulus GPa			Poisson’s Ratio			Shear Modulus GPa		
	$E_{X}$	$E_{Y}$	$E_{Z}$	$\nu_{XY}$	$\nu_{XZ}$	$\nu_{YZ}$	$G_{XY}$	$G_{XZ}$	$G_{YZ}$
Predicted value (tension)	37.0	33.6	44.6	0.37	0.09	0.11	12.2	3.3	2.5
Experimental value (tension)	38.3	-	40.5	0.28	0.09	-	12.6	3.4	-
Predicted value (compression)	22.3	20.5	21.1	0.38	0.12	0.15	-	-	-
Experimental value (compression)	20.9	-	22.1	0.28	0.09	-	-	-	-

Note: Angle mark *Z* represents the fiber rod axial direction, Angle mark *X* and *Y* represent the transverse direction. Notice the difference between the direction sign here and the direction sign of the meso component.

**Table 6:** Coefficient of thermal expansion at room temperature.

Item	Axial Coefficient of Thermal Expansion, 10^−6^/K	Radial Coefficient of Thermal Expansion, 10^−6^/K
Predicted value	0.147	−0.477
Experimental value	0.145	−0.50
